# The TyG Index Mediates Air-Pollution-Associated Chronic Kidney Disease Incidence in HIV/AIDS Patients: A 20-Year Cohort Study

**DOI:** 10.3390/toxics13080669

**Published:** 2025-08-08

**Authors:** Xiaoxia Liu, Xiuli Zhao, Lu Ye, Chengfeng Hu, Zhihao Xie, Jianan Ma, Xia Wang, Wei Liang

**Affiliations:** 1 Zhenjiang Center for Disease Control and Prevention, 9# South Huangshan Road, Zhenjiang 212002, China; 2 School of Public Health, Faculty of Medicine, Yangzhou University, 136# Jiangyang Middle Road, Yangzhou 225000, China; 3 Wuhan Center for Disease Control and Prevention, 288# Machang Road, Wuhan 430024, China

**Keywords:** air pollutants, chronic kidney disease, HIV/AIDS, TyG, eGFR

## Abstract

Ambient air pollutants (APs) are associated with increased chronic kidney disease (CKD) risk in general populations, but their renal impact on HIV/AIDS patients remains understudied. This dynamic cohort included 7981 HIV/AIDS patients without baseline kidney disease from Wuhan and Zhenjiang, followed every 6 months with fasting blood tests to assess the triglyceride-glucose (TyG) index and estimated glomerular filtration rate (eGFR). Monthly average exposures to six APs were estimated from geocoded residential addresses. Modified Poisson regression models were used to assess associations between cumulative AP exposure and CKD incidence, with mediation analysis conducted to explore the potential role of the TyG index. Weighted quantile sum regression was applied to evaluate the joint effects of six APs. During the follow-up period, 168 new cases of CKD were identified. Each interquartile range increase in PM_2.5_, PM_10_, and SO_2_ corresponded to a 16.5%, 18.9%, and 9.7% higher CKD risk, respectively, with the TyG index mediating 10.21%, 9.16%, and 5.14% of these associations. PM_2.5_ demonstrated the highest attribution weight (44.4%) for CKD risk elevation in mixed-exposure models. Chronic ambient AP exposure, particularly PM_2.5_, synergistically elevates CKD risk in HIV/AIDS patients with glucolipid dysregulation potentially being involved, necessitating targeted air quality policies to mitigate AP impacts on this vulnerable population.

## 1. Introduction

Chronic kidney disease (CKD), an irreversible chronic condition marked by persistent anomalies in kidney structure and function, poses a significant health challenge worldwide [1]. Global disease burden research highlights a rapid increase in the incidence and mortality rates of CKD worldwide [2], rendering it as a significant contributor to global mortality [3]. Since 1990, the worldwide prevalence of CKD among adults has surged by 23.9, affecting over 697 million people in 2019. Notably, China reported the highest patient count, reaching 132 million in 2020 [4]. Growing evidence suggests that CKD risk factors involve genetics, environment, underlying conditions, immune status, and lifestyle behaviors [5,6]. Recently, there has been increasing attention given to the impact of air pollutants (APs) on kidney disease [7,8].

In 2017, approximately 122 million cases of CKD worldwide were reportedly attributed to fine particulate matter (PM_2.5_) exposure [9]. Multiple studies have elucidated that prolonged exposure to APs among healthy populations is linked to a decline in renal function, consequently heightening the risk of CKD [10,11]. A cohort study conducted in the United States also found that heightened exposure to elevated concentrations of PM_10_, nitrogen dioxide (NO_2_), and carbon monoxide (CO) was correlated with an augmented risk of CKD and end-stage renal diseases [8]. HIV/AIDS patients, as a group with compromised immune systems, might be more susceptible to APs [12,13]. To date, only one study has investigated the impact of ambient particulate matter exposure on kidney function in individuals living with HIV/AIDS [14], reporting that long-term PM_2.5_ exposure was positively associated with incident CKD (HR: 1.599, 95% CI: 1.431–1.787 per 14.1 µg/m^3^ increment). However, this work only included particulate pollutants, excluding gaseous pollutants and multi-pollutant combined effects. Our study addresses these gaps by incorporating both particulate and gaseous pollutants and applying mixture modeling to assess multi-pollutant joint effects.

The triglyceride-glucose (TyG) index, a reliable and widely used surrogate marker of insulin resistance [15], has recently gained attention in the context of chronic disease risk prediction, including CKD [16]. Emerging evidence suggests that air pollution, particularly PM_2.5_ exposure, may contribute to systemic insulin resistance through oxidative stress and inflammation [17]. Given that insulin resistance is a recognized contributor to renal dysfunction, the TyG index may offer valuable insight into the biological mechanisms linking air pollution exposure and CKD development.

Recognizing the heightened vulnerability of individuals with HIV/AIDS to environmental stressors, this study investigated the association between exposure to both particulate and gaseous APs and the risk of CKD in this population. Particular emphasis was placed on the potential mediating role of the TyG index and the impact of multi-pollutant mixed exposure. Our findings can deliver actionable evidence for prioritizing particulate matter mitigation policies to alleviate air-pollution-associated CKD disparities in immunocompromised populations.

## 2. Materials and Methods

### 2.1. Study Population

**Figure 1:**
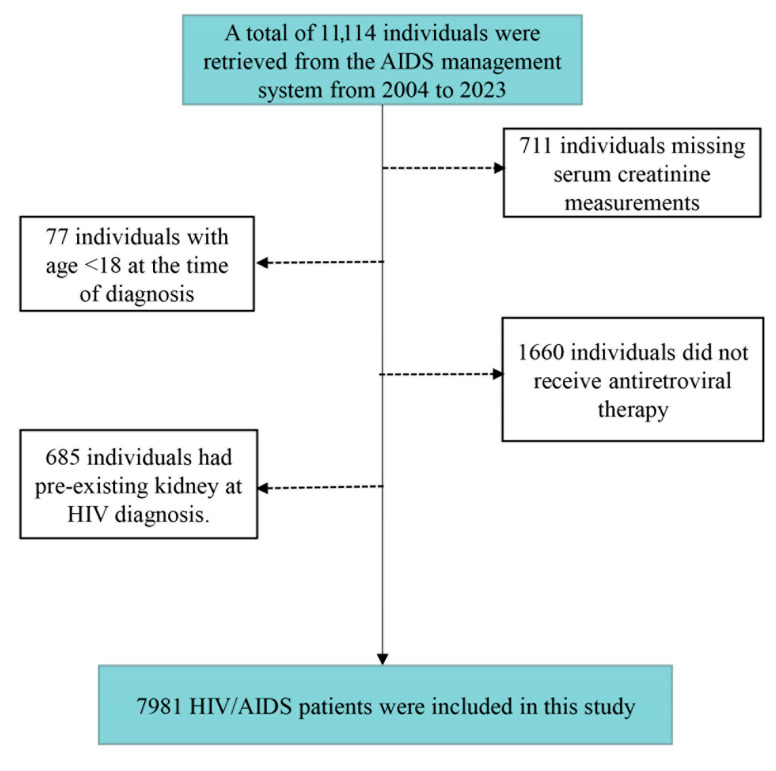
Flowchart for inclusion of study subjects.

Data for this study were obtained from the HIV/AIDS Comprehensive Response Information Management System (CRIMS), maintained by the Centers for Disease Control and Prevention (CDCs) in Wuhan and Zhenjiang, covering the period from 1 January 2004, to 31 December 2023. Throughout this duration, information was collected from 11,114 HIV/AIDS patients through CRIMS, encompassing basic personal information, records of each follow-up visit, and treatment program. A total of 3133 HIV/AIDS patients were excluded from the analysis due to having an age under 18 at diagnosis (*n* = 77), a lack of antiretroviral therapy (*n* = 1660), missing serum creatinine (Scr) measurements (*n* = 711), or having pre-existing kidney disease at the time of HIV diagnosis (*n* = 685) (Figure 1). We included 7981 HIV/AIDS patients without CKD at baseline in the follow-up to assess the subsequent development of CKD. This study was granted approval by the ethics committee of Wuhan CDC (WHCDCIRB-K-2022027).

### 2.2. Biomarker Measurements

During each follow-up visit, conducted at six-month intervals, medical personnel collected venous blood samples (5 mL without anticoagulant) from participants after at least 8 h of fasting. Blood samples were centrifuged at 3000 revolutions per minute for 10 min to isolate serum. Serum levels of Scr, triglyceride (TG), and fasting blood glucose (FBG) were acquired using an auto-analyzer. The estimated glomerular filtration rate (eGFR) for each HIV/AIDS patient was computed utilizing the CKD Epidemiology Collaboration (CKD-EPI) equation [18]. CKD is defined as a less than 60 mL/min/1.73 m^2^ eGFR. The TyG index is computed using the following formula: TyG index = ln [TG (mg/dL) × FBG (mg/dL)/2].

### 2.3. Exposure Assessment

Daily AP concentration was obtained from the China High Air Pollutants dataset (CHAP, https://weijing-rs.github.io/product.html), which employs space–time extremely randomized tree models to generate full-coverage pollutant estimates. The dataset provides concentrations of particulate matter (PM_1_, PM_2.5_, PM_10_) and ozone (O_3_) at a spatial resolution of 1 km × 1 km, while NO_2_, sulfur dioxide (SO_2_), and CO are available at a resolution of 10 km × 10 km [19,20,21,22,23,24]. Individual exposure was estimated by geocoding each participant’s residential address, which had been de-identified by professional staff at the CDC to ensure privacy protection. Pollutant concentrations from the nearest grid cell were then assigned to each participant’s location using Google Maps.

### 2.4. Covariates

We selected covariates based on previous studies that have investigated the relationship between APs and renal function [11,14,25]. The demographic information gathered encompassed age, sex, body mass index (BMI), education level (junior secondary education, senior secondary education, higher education), marital status (unmarried, married, divorced), and work types (outdoor worker, indoor worker, unknown). Details about habits and health conditions covered infection status (HIV, AIDS), smoking status, and alcohol consumption. Treatment medications were categorized based on the presence or absence of tenofovir disoproxil fumarate (TDF) (no, yes), which could potentially lead to renal impairment. TG and TC levels measured during each follow-up were taken into account, alongside the distinction between warm (May to October) and cool (November to April) seasons at the time of follow-up, serving as confounding factors.

### 2.5. Statistical Analyses

Modified Poisson regression models were employed to assess the association between cumulative AP exposure and incident CKD. Individual cumulative exposure was defined as the average pollutant concentration experienced from baseline to either the end of follow-up or CKD onset. Risk ratios (RRs) and 95% confidence intervals (CIs) were calculated to quantify the effect of AP exposure per interquartile range (IQR) increase on CKD risk. In our analysis, two regression models were executed. Model 1 incorporated adjustments for age, sex, BMI, education level, marital status, work types, smoking status, alcohol consumption, and season. Model 2, extending model 1, encompassed additional adjustments for infection status, use of TDF, TC, and TG. Exposure–response relationships between AP concentrations and the RRs of CKD were modeled using restricted cubic splines with four degrees of freedom.

Weighted quantile sum (WQS) regression is a method for analyzing exposure mixtures, which accounts for correlations among components while estimating both the overall effect of the mixture and the relative contribution of each component. In our study, WQS regression models with 5000 bootstrap iterations were employed to comprehensively evaluate the association of mixed exposure to six APs with CKD incidence. The pollutant concentrations underwent standardization into quartiles, leading to the development of a composite exposure index. This index facilitated the assessment of mixed exposure effects through the derivation of a WQS index in both positive and negative directions. We could also infer the contribution of each pollutant within the mixed exposure model, thus identifying the primary pollutants.

Mediation analyses followed a two-step regression framework and used the mediation package with a nonparametric bootstrap approach (5000 resamples) to delineate pathways linking air pollution exposure to CKD risk and quantify the mediating role of the TyG index. Specifically, we first modeled the effect of air pollution on TyG (mediator) using a general linear model, then assessed the associations of air pollution and TyG with CKD (outcome) via modified Poisson regression. The total effect was decomposed into (1) the direct effect (DE), representing exposure’s impact on outcome independent of the mediator; and (2) the indirect effect (IE), mediated through TyG index alterations. Mediation proportions were calculated as IE/(DE + IE), with bias-corrected 95% confidence intervals derived from bootstrap distributions.

Three sensitivity analyses were performed to check the robustness of our main findings. (1) We evaluated the association between AP exposure and the risk of incident CKD after excluding individuals with a history of smoking or alcohol consumption, in order to reduce potential confounding effects from lifestyle factors. (2) Cox proportional hazards models were employed to re-evaluate the association between AP exposure and incident CKD. (3) To account for multiple comparisons, we applied the False Discovery Rate (FDR) correction to the *p*-values derived from pollutant-specific models. Associations with FDR-adjusted *p*-values < 0.05 were considered statistically significant. All statistical analyses were performed using R software (Version 4.1.3). The R packages used in this study include “mediation” and “gWQS”.

## 3. Results

### 3.1. Description of the Study Sample and Exposure

The demographic profile of the 7981 HIV/AIDS patients is presented in Table 1. This study included individuals with an average age of 38.7 ± 14.6 years and a mean BMI of 22.0 ± 3.81 kg/m^2^. The main subjects were male (89.6%), indoor workers (50.6%), and unmarried (52.2%). In addition, 63.1% of all participants were diagnosed with HIV, and 61.0% had used TDF drugs. Most participants had never smoked (68.7%) or consumed alcohol (71.0%). Table S1 shows the distribution of AP concentrations during the follow-up period. During the follow-up period, IQRs of PM_2.5_, PM_10_, SO_2_, CO, NO_2_, and O_3_ were 13.2 μg/m^3^, 20.2 μg/m^3^, 2.9 μg/m^3^, 0.2 mg/m^3^, 13.2 μg/m^3^, and 16.7 μg/m^3^, respectively.

### 3.2. Associations Between AP Exposure and Incidence of CKD

**Figure 2:**
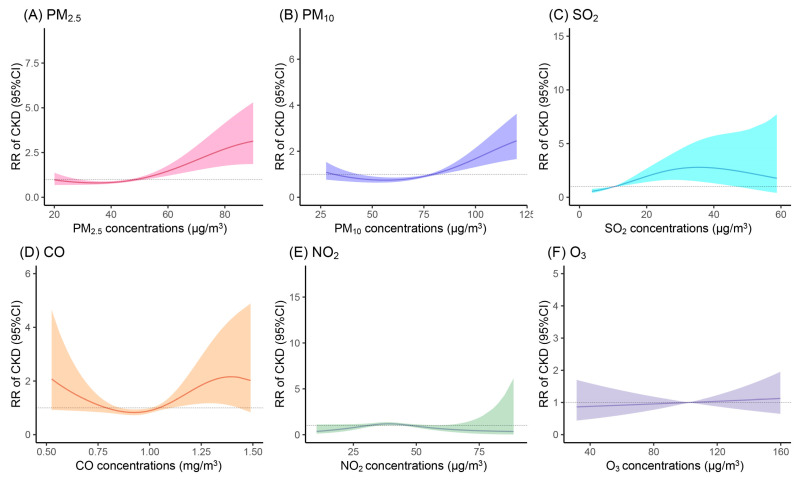
Exposure–response curves for the associations between air pollutant exposure and relative risk of CKD. Models were adjusted for age, sex, BMI, education level, marital status, work types, smoking status, alcohol consumption, season, infection status, use of TDF, TC, and TG. Abbreviations: PM_2.5_, the aerodynamic diameter is less than 2.5 μg/m^3^; PM_10_, the aerodynamic diameter is less than 10 μg/m^3^; SO_2_, sulfur dioxide; CO, carbon monoxide; NO_2_, nitrogen dioxide; O_3_, ozone.

By 31 December 2023, 168 new cases of CKD were identified among the 7891 HIV/AIDS patients under follow-up. In fully adjusted models, chronic exposure to PM_2.5_, PM_10_, and SO_2_ was significantly associated with increased risk of CKD. Specifically, each IQR increase in PM_2.5_ was associated with a 16.5% higher risk (RR: 1.165, 95% CI: 1.030, 1.317), in PM_10_ with an 18.9% higher risk (RR: 1.189, 95% CI: 1.016, 1.390), and in SO_2_ with a 9.7% higher risk (RR: 1.097, 95% CI: 1.039, 1.159) (Table 2). However, concentrations of CO, NO_2_, and O_3_ did not exhibit statistically significant associations with CKD incidence. Penalized spline analyses demonstrated near-linear increases in CKD risk with rising PM_2.5_, PM_10_, and O_3_ concentrations. In contrast, SO_2_ and NO_2_ showed positive associations at lower exposure levels that attenuated or inverted at higher concentrations, while CO exhibited an S-shaped exposure–response trajectory (Figure 2).

### 3.3. Mixed AP Exposure on CKD Incidence via Weighted Quantile Sum Modeling

**Figure 3:**
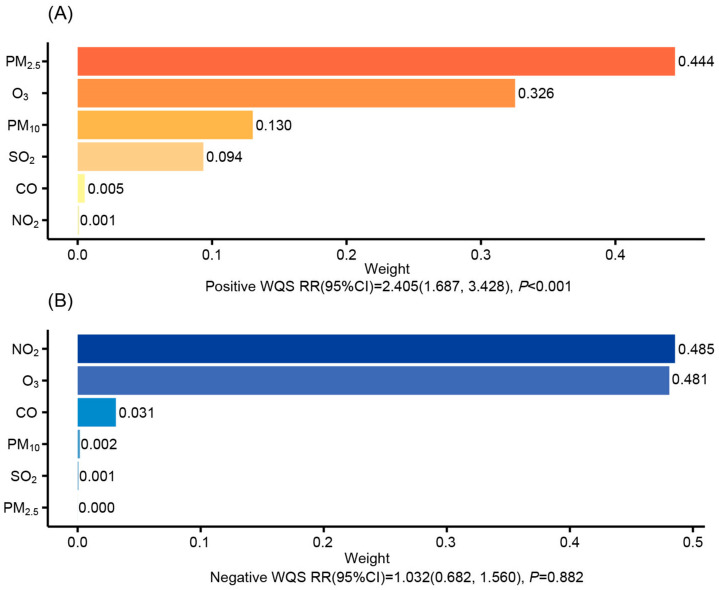
The weights of six air pollutants with the risk of CKD based on WQS regression. Models were adjusted for age, sex, BMI, education, marital status, work types, smoking status, alcohol consumption, season, infection status, use of TDF, TC, and TG. (**A**) Positive-direction WQS Regression; (**B**) Negative-direction WQS Regression. Abbreviations: PM_2.5_, the aerodynamic diameter is less than 2.5 μg/m^3^; PM_10_, the aerodynamic diameter is less than 10 μg/m^3^; SO_2_, sulfur dioxide; CO, carbon monoxide; NO_2_, nitrogen dioxide; O_3_, ozone.

The WQS regression model unveiled a positive correlation between the WQS index of mixed AP exposure and incident CKD (RR: 2.405, 95% CI: 1.687, 3.428). Notably, PM_2.5_ emerged as the dominant contributor, accounting for 44.4% of the overall mixture weight, followed by O_3_ (32.6%), PM_10_ (13.0%), and SO_2_ (9.4%) (Figure 3A). Conversely, the negative-direction WQS index showed no significant inverse relationship with CKD risk (RR:1.032, 95%CI:0.682, 1.560) (Figure 3B).

### 3.4. Mediation Analyses and Sensitivity Analyses

Mediation analysis revealed that the TyG index partially mediated the association between cumulative exposure to AP and CKD risk, accounting for 10.21% (95% CI:1.70%, 32.06%) for PM_2.5_, 9.16% (95% CI:1.54%, 30.95%) for PM_10_, and 5.14% (95% CI:0.75%, 13.93%) for SO_2_ (Table 3). In sensitivity analyses, consistent associations between AP exposure and CKD risk were observed after excluding individuals with a history of smoking or alcohol consumption (Table S2). Similarly, the associations between AP exposure and CKD risk remained robust when evaluated using Cox proportional hazards models (Table S3), with consistent results observed after applying FDR correction (Table S4).

## 4. Discussion

To our knowledge, this is the first large-scale longitudinal study examining the association between exposure to six ambient APs and CKD in HIV/AIDS patients. Our findings demonstrated that chronic exposure to PM_2.5_, PM_10_, and SO_2_ was significantly associated with an elevated risk of CKD, with the TyG index partially mediating these associations. Among the six APs analyzed in mixture exposure models, PM_2.5_ emerged as the most significant contributor to elevated CKD risk.

Our study revealed that chronic exposure to PM_2.5_, PM_10_, and SO_2_ was related to a higher incidence of CKD, which is in agreement with the results of other studies in China. A study conducted on 72,425 Chinese adults observed that each 10 ug/m^3^ increase in long-term PM_2.5_ exposure was linked with a higher prevalence of CKD (OR, 1.71, 95CI: 1.58–1.85) [26]. A meta-analysis of 32 studies indicated that for every 10 μg/m^3^ increase in PM_2.5_, PM_10_, NO_2_, and NO_X_, the risk CKD increased by 42%, 20%, 7%, and 7%, respectively. Additionally, an increase of 1 ppb SO_2_ led to a 7% rise in CKD risk, while a 0.1 ppm rise in CO showed a 3% increase [27]. However, some studies did not find associations between long-term exposure to PM_2.5_, SO_2_, and NO_2_ and the risk of CKD [28,29]. The existing findings are inconsistent due to variations among different population groups, disparities in exposure levels, and differences in the duration of exposure periods. 

In our analysis, we found no significant associations between CO, NO_2_, and O_3_ and CKD risk, which may indicate a true lack of effect in our population. Biologically, particulate matter more directly induces systemic oxidative stress, inflammation, and endothelial dysfunction, key processes in CKD development. In contrast, the mechanisms by which NO_2_ and O_3_ impact renal function are less clear and likely weaker. Additionally, their high reactivity, shorter atmospheric lifetimes, and distinct exposure patterns may limit their renal toxicity compared to particulate matter. The null findings may be attributable to the limited statistical power caused by smaller effect sizes and the relatively low ambient concentrations of these gaseous pollutants. Further studies with larger samples and improved exposure assessments are warranted to clarify the roles of these gaseous pollutants in CKD.

Notably, this study further revealed through WQS regression that among the six mixed APs, PM_2.5_ contributed the most to CKD risk, significantly more than PM_10_ and SO_2_. This finding may be attributed to the submicron particle size (≤2.5 μm) of PM_2.5_ and its enriched toxic components (e.g., transition metals, polycyclic aromatic hydrocarbons) and HIV-related pathophysiology, which facilitate its deep penetration through the alveolar barrier and induction of systemic oxidative stress and renal vascular endothelial damage [30,31]. HIV-related chronic immune activation and antiretroviral-therapy-associated mitochondrial dysfunction amplify these effects, driving tubulointerstitial fibrosis and vascular damage—processes further exacerbated by the inherent immunometabolic vulnerabilities of the HIV population [32]. The dominance of PM_2.5_ over gas-phase pollutants highlights particle-bound toxins as key drivers of renal injury in this vulnerable population, underscoring the need for HIV-specific pollution risk assessments.

Our analysis revealed that the TyG index may be involved in the association between AP exposure and increased CKD risk. The TyG index, a novel marker for insulin resistance, is linked to PM_2.5_-induced pulmonary oxidative stress, as evidenced in animal models [33]. Additionally, studies have identified a relationship between PM_2.5_ exposure and elevated insulin resistance markers in humans [34], suggesting AP exposure may be linked to insulin resistance, with the TyG index potentially serving as an indicative marker. Presently, the TyG index is acknowledged as a predictive tool for CKD onset [35], with recent evidence highlighting a significant correlation between the TyG index and CKD in individuals with type 2 diabetes [36]. PM_2.5_ may induce systemic oxidative stress and chronic low-grade inflammation, impairing insulin signaling and lipid metabolism. These effects can elevate circulating glucose and triglyceride levels—key components of the TyG index—and contribute to glomerular injury, endothelial dysfunction, and renal microvascular damage. However, given the observational nature of the data and potential unmeasured confounding effects, these findings should be interpreted as exploratory and not as conclusive evidence of causality. While the mediation proportion was relatively small, this is expected given the multifactorial etiology of CKD. The TyG index may represent only one of several parallel metabolic pathways involved, and even partial mediation provides useful insight into the biological processes linking air pollution to kidney impairment.

The potential mechanisms behind renal damage associated with AP exposure are not yet fully understood. Prior research has indicated that elevated AP levels may activate cytokines or oxidative agents in the respiratory tract, leading to inflammation. These agents can then circulate through the body, causing damage to distant organs, including the kidneys [37,38]. Studies involving rodent models and in vitro cellular assays have demonstrated that inhibiting inflammatory pathways can mitigate kidney damage resulting from PM_2.5_ exposure [39]. Moreover, individuals with pre-existing health conditions, such as HIV/AIDS patients, are already in an inflammatory state [40,41], and APs may contribute to renal damage by exacerbating the level of inflammation in patients. Meanwhile, an AP-induced high inflammatory state in HIV/AIDS patients can cause immune cells to be destroyed and can weaken immune function [12,42]. Given that HIV/AIDS patients are a vulnerable group with compromised immune systems, they exhibit heightened susceptibility to the hazards posed by APs. This vulnerability can impair their infection-fighting capabilities, potentially leading to enhanced viral replication and accelerated disease progression that affects the immune system, further exacerbating PM-related kidney damage.

There were some limitations in this study. Firstly, the exposure data used for APs were evaluated using satellite-based spatiotemporal models, calculating outdoor pollutant concentrations. This method overlooked individual indoor exposure levels, which could potentially introduce measurement biases. Secondly, information on individual-level socioeconomic status, such as income or education, was not available, which may introduce residual confounding. However, given that all participants were recruited from the same public health system and geographic area, the variability in socioeconomic status may be limited. Thirdly, while we adjusted for TDF use, detailed data on the duration and type of other ARTs were not available. Due to the complexity and variability of ART regimens, further adjustment was not feasible. Nonetheless, any potential confounding from unmeasured ART components is unlikely to be strongly associated with air pollution exposure and thus would likely result in non-differential bias. Fourthly, in this study, CKD diagnosis was based on a single eGFR measurement below 60 mL/min/1.73 m^2^, rather than the clinical standard of two measurements 90 days apart, due to irregular follow-up intervals. While this approach deviates from clinical diagnostic criteria, it is a common practice in environmental epidemiology studies investigating the association between air pollution and kidney outcomes. We acknowledge that relying on a single eGFR measurement may overestimate CKD incidence by including cases of transient or reversible kidney function decline. However, any potential misclassification is likely to be non-differential with respect to air pollution exposure, which would bias the estimates toward the null rather than exaggerate the observed associations. Therefore, this approach, although suboptimal from a clinical standpoint, remains appropriate for etiological inference in population-based environmental research. Finally, our study was conducted in Wuhan and Zhenjiang, with findings potentially constrained by regional-specific factors (e.g., pollutant profiles, demographics). Thus, external validity in other regions requires confirmation via future studies.

## 5. Conclusions

Our study indicates that exposure to APs, particularly particulate matter, is associated with diminished renal function and an increased incidence of CKD in HIV/AIDS patients. The TyG index, as a marker of glucolipid metabolism, may offer potential insights into the metabolic pathways linking particulate matter exposure to renal damage in this population. Urgent policies are necessary to mitigate AP-induced kidney damage in vulnerable populations.

## Figures and Tables

**Table 1:** Descriptive characteristics of study subjects at baseline.

Variables	Total (N = 7981)
Age, years, (Mean ± SD)	38.7 ± 14.6
BMI, kg/m^2^ (Mean ± SD)	22.0 ± 3.81
Sex, *n* (%)	
Male	7152 (89.6)
Female	829 (10.4)
Infection status	
HIV	5033 (63.1)
AIDS	2948 (36.9)
Education level, *n* (%)	
Junior secondary education	2341 (29.3)
Senior secondary education	2078 (26.0)
Higher education	3562 (44.6)
Work types, *n* (%)	
Outdoor worker	3358 (42.1)
Indoor worker	4042 (50.6)
Unknown	581 (7.3)
Marital status, *n* (%)	
Unmarried	4165 (52.2)
Married	2219 (27.8)
Divorced	1597 (20.0)
Smoking status, *n* (%)	
Never	5486 (68.7)
Former	566 (7.1)
Current	1929 (24.2)
Alcohol consumption, *n* (%)	
Never	5670 (71.0)
Former	549 (6.9)
Current	1762 (22.1)
Use of TDF, *n* (%)	
No	3116 (39.0)
Yes	4865 (61.0)
Serum creatinine, μmol/L (Mean ± SD)	71.2 ± 14.0
Cholesterol, mmol/L (Mean ± SD)	4.04 ± 0.881
Triglyceride, mmol/L (Mean ± SD)	1.82 ± 2.00
Fasting blood glucose, mmol/L (Mean ± SD)	5.74 ± 1.41

Abbreviations: SD, standard deviation; BMI, body mass index; HIV, human immunodeficiency virus; AIDS, acquired immunodeficiency syndrome; TDF, tenofovir disoproxil fumarate.

**Table 2:** Associations between air pollutant exposure and CKD in HIV/AIDS patients.

Outcomes	Pollutants	Model 1 ^a^	Model 2 ^b^
RR of CKD (95%CI)	PM_2.5_	1.212 (1.078, 1.363)	1.165 (1.030, 1.317)
	PM_10_	1.244 (1.069, 1.448)	1.189 (1.016, 1.390)
	SO_2_	1.128 (1.074, 1.185)	1.097 (1.039, 1.159)
	CO	1.090 (0.916, 1.297)	1.040 (0.876, 1.234)
	NO_2_	0.975 (0.811, 1.172)	0.950 (0.788, 1.146)
	O_3_	1.024 (0.886, 1.184)	1.035 (0.889, 1.205)

^a^ Model 1 was adjusted for age, sex, BMI, education, marital status, work types, smoking status, alcohol consumption, and season. ^b^ Model 2 was adjusted for age, sex, BMI, education, marital status, work types, smoking status, alcohol consumption, season, infection status, use of TDF, TC, and TG. All effect estimates for air pollutants correspond to an IQR increment: PM_2.5_ (13.2 μg/m^3^), PM_10_ (20.2 μg/m^3^), SO_2_ (2.9 μg/m^3^), CO (0.2 mg/m^3^), NO_2_ (13.2 μg/m^3^), and O_3_ (16.7 μg/m^3^). Abbreviations: PM_2.5_, the aerodynamic diameter is less than 2.5 μg/m^3^; PM_10_, the aerodynamic diameter is less than 10 μg/m^3^; SO_2_, sulfur dioxide; CO, carbon monoxide; NO_2_, nitrogen dioxide; O_3_, ozone.

**Table 3:** Mediation analyses of TyG index on the associations between air pollutants and CKD.

Exposure	Direct Effect (95% CI)	Indirect Effect (95% CI)	Proportion Mediated (95% CI)
PM_2.5_	1.0021 (1.0008, 1.0028)	1.0003 (1.0000, 1.0005)	10.21% (1.70%, 32.06%)
PM_10_	1.0020 (1.0008, 1.0027)	1.0002 (1.000, 1.0005)	9.16% (1.54%, 30.94%)
SO_2_	1.0018 (1.0010, 1.0024)	1.0001 (1.0000, 1.0002)	5.14% (0.75%, 13.93%)
CO	0.9996 (0.9942, 1.0015)	1.0008 (1.0001, 1.0019)	32.93% (−574.65%, 519.90%)
NO_2_	0.9974 (0.9888, 1.0021)	1.0008 (1.0002, 1.0016)	−17.41% (−383.13%, 433.09%)
O_3_	1.0005 (0.9960, 1.0019)	0.9996 (0.9990, 0.9999)	−14.6% (−296.35%, 310.96%)

All effect estimates for air pollutants correspond to an IQR increment: PM_2.5_ (13.2 μg/m^3^), PM_10_ (20.2 μg/m^3^), SO_2_ (2.9 μg/m^3^), CO (0.2 mg/m^3^), NO_2_ (13.2 μg/m^3^), O_3_ (16.7 μg/m^3^). Abbreviations: PM_2.5_, the aerodynamic diameter is less than 2.5 μg/m^3^; PM_10_, the aerodynamic diameter is less than 10 μg/m^3^; SO_2_, sulfur dioxide; CO, carbon monoxide; NO_2_, nitrogen dioxide; O_3_, ozone.

## Data Availability

Data will be made available on request.

## Supplementary Materials

The following supporting information can be downloaded at: https://www.mdpi.com/article/10.3390/toxics13080669/s1, Table S1. Distribution of air pollutant concentrations during the follow-up period; Table S2. Associations between air pollutant exposure and incident chronic kidney disease after excluding individuals with a history of smoking or alcohol consumption; Table S3. Re-evaluating the associations between air pollutant exposures and chronic kidney disease using Cox proportional hazards models; Table S4. Associations between air pollutants and CKD risk with FDR correction in HIV/AIDS patients.

## Abbreviations

The following abbreviations are used in this manuscript:

PM_2.5_	the aerodynamic diameter is less than 2.5 μg/m^3^;
PM_10_	the aerodynamic diameter is less than 10 μg/m^3^
SO_2_	sulfur dioxide
CO	carbon monoxide
NO_2_	nitrogen dioxide
O_3_	ozone
CKD	chronic kidney disease
AP	air pollutant
TyG	triglyceride-glucose
CRIMS	Comprehensive Response Information Management System
CDC	Center for Disease Control and Prevention
TG	triglyceride
WQS	weighted quantile sum
FBG	fasting blood glucose
Scr	serum creatinine
eGFR	estimated glomerular filtration rate
TDF	tenofovir disoproxil fumarate
RRs	risk factors
CIs	confidence intervals
IQR	interquartile range
CKD	Epidemiology Collaboration (CKD-EPI)
BMI	body mass index
