# Supplementary material for: The TyG Index Mediates Air-Pollution-Associated Chronic Kidney Disease Incidence in HIV/AIDS Patients: A 20-Year Cohort Study
Source: Toxics. 2025 Aug 8;13(8):669. doi: 10.3390/toxics13080669 (PMC12389780; doi:10.3390/toxics13080669)
Supplement: Supplementary file 1 [file toxics-13-00669-s001.zip › toxics-3769287-supplementary.pdf]

The TyG Index Mediates Air Pollution-Associated Chronic Kidney Disease Incidence in HIV/AIDS patients: A 20-Year Cohort Study

Table content

Table S1 Distribution of air pollutants concentrations during the follow-up Period.

Table S2 Associations between air pollutant exposure and incident chronic kidney disease after excluding individuals with a history of smoking or alcohol consumption.

Table S3 Re-evaluating the associations between air pollutant exposures and chronic kidney disease using Cox proportional hazards models.

Table S4 Associations between air pollutants and CKD risk with FDR correction in HIV/AIDS patients.

Table S1 Distribution of air pollutants concentrations during the follow-up Period.

Exposure (unit)	Minimum	P_{25}	median	P_{75}	Maximum	Mean	IQR
$PM_{2.5}$(μg/m^3)	2.2	37.4	44.1	50.6	175.1	44.3	13.2
PM_{10} (μg/m^3)	1.2	63.7	73	83.9	235.3	73.3	20.2
SO_2 (μg/m^3)	3.7	8.5	9.7	11.4	78.7	10.8	2.9
CO (mg/m^3)	0.5	0.9	1.0	1.1	2.2	1.0	0.2
NO_2 (μg/m^3)	9.4	34.4	41.9	47.6	98.6	41.1	13.2
O_3 (μg/m^3)	31.5	93.7	101.3	110.4	175.8	102.2	16.7

Note: P_{25}, the 25th quantile; P75, the 75th quantile; IQR, interquartile ranges; $PM_{2.5}$,the aerodynamic diameter is less than 2.5μg/m^3; PM_{10},the aerodynamic diameter is less than 10μg/m^3; SO_2, sulfur dioxide; CO, carbon monoxide; NO_2, nitrogen dioxide; O_3, ozone.

Table S2 Associations between air pollutant exposure and incident chronic kidney disease after excluding individuals with a history of smoking or alcohol consumption.

Outcomes	Pollutants	Model 1[a]	Model 2[b]
RR of CKD (95% CI)	$PM_{2.5}$	1.431 (1.185, 1.728)	1.345 (1.104, 1.638)
	PM_{10}	1.564 (1.255, 1.949)	1.461 (1.170, 1.824)
	SO_2	1.254 (1.183, 1.328)	1.206 (1.126, 1.291)
	CO	1.123 (0.883, 1.429)	1.092 (0.863, 1.381)
	NO_2	1.045 (0.819, 1.333)	1.043 (0.813, 1.337)
	O_3	0.988(0.814, 1.199)	0.987 (0.807, 1.207)

[a]Model 1 were adjusted for age, sex, BMI, education level, marital status, work types, smoking status, alcohol consumption, and season. [b]Model 2 were adjusted for age, sex, BMI, education level, marital status, work types, smoking status, alcohol consumption, season, infection status, TDF, TC, and TG. All effect estimates for air pollutants correspond to an IQR increment: $PM_{2.5}$ (13.2μg/m³), PM_{10} (20.2 μg/m³), SO_2 (2.9μg/m³), CO (0.2mg/m³), NO_2 (13.2μg/m³), O_3 (16.7μg/m³). Note: $PM_{2.5}$,the aerodynamic diameter is less than 2.5μg/m³; PM_{10},the aerodynamic diameter is less than 10μg/m³; SO_2, sulfur dioxide; CO, carbon monoxide; NO_2, nitrogen dioxide; O_3, ozone.

Table S3 Re-evaluating the associations between air pollutant exposures and chronic kidney disease using Cox proportional hazards models.

Outcomes	Pollutants	Model 1[a]	Model 2[b]
HR of CKD (95% CI)	$PM_{2.5}$	1.317 (1.079, 1.608)	1.369 (1.106, 1.695)
	PM_{10}	1.219 (1.015, 1.464)	1.248 (1.028, 1.514)
	SO_2	1.239 (1.135, 1.352)	1.243 (1.131, 1.365)
	CO	1.017 (0.801, 1.29)	1.010 (0.789, 1.292)
	NO_2	0.910 (0.686, 1.207)	0.913 (0.688, 1.213)
	O_3	1.166 (0.866, 1.568)	1.170 (0.865, 1.582)

[a]Model 1 were adjusted for age, sex, BMI, education level, marital status, work types, smoking status, alcohol consumption, and season. [b]Model 2 were adjusted for age, sex, BMI,

education level, marital status, work types, smoking status, alcohol consumption, season, infection status, TDF, TC, and TG. All effect estimates for air pollutants correspond to an IQR increment: $PM_{2.5}$ (13.2μg/m³), PM_{10} (20.2 μg/m³), SO_2 (2.9μg/m³), CO (0.2mg/m³), NO_2 (13.2μg/m³), O_3 (16.7μg/m³). Note: $PM_{2.5}$,the aerodynamic diameter is less than 2.5μg/m³; PM_{10}, the aerodynamic diameter is less than 10μg/m³; SO_2, sulfur dioxide; CO, carbon monoxide; NO_2, nitrogen dioxide; O_3, ozone.

Table S4 Associations between air pollutants and CKD risk with FDR correction in HIV/AIDS patients.

Outcomes	Pollutants	Model 1[a]	*FDR*	Model 2[b]	*FDR*
RR of CKD (95%CI)	$PM_{2.5}$	1.212 (1.078, 1.363)	0.00126	1.165 (1.030, 1.317)	0.04500
	PM_{10}	1.244 (1.069, 1.448)	0.00485	1.189 (1.016, 1.390)	0.04984
	SO_2	1.128 (1.074, 1.185)	0.00000	1.097 (1.039, 1.159)	0.00522
	CO	1.090 (0.916, 1.297)	0.33190	1.040 (0.876, 1.234)	0.65991
	NO_2	0.975 (0.811, 1.172)	0.78519	0.950 (0.788, 1.146)	0.65991
	O_3	1.024 (0.886, 1.184)	0.74916	1.035 (0.889, 1.205)	0.65991

[a]Model 1 were adjusted for age, sex, BMI, education, marital status, work types, smoking status, alcohol consumption, and season. [b]Model 2 were adjusted for age, sex, BMI, education, marital status, work types, smoking status, alcohol consumption, season, infection status, use of TDF, TC, and TG. All effect estimates for air pollutants correspond to an IQR increment: $PM_{2.5}$ (13.2μg/m³), PM_{10} (20.2 μg/m³), SO_2 (2.9μg/m³), CO (0.2mg/m³), NO_2 (13.2μg/m³), O_3 (16.7μg/m³). Abbreviations: $PM_{2.5}$, the aerodynamic diameter is less than 2.5μg/m³; PM_{10}, the aerodynamic diameter is less than 10μg/m³; SO_2, sulfur dioxide; CO, carbon monoxide; NO_2, nitrogen dioxide; O_3, ozone; FDR, false discovery rate.
